# Annual Meeting of the Governors and General Council

**Published:** 1913-05-03

**Authors:** 


### KING EDWARD'S HOSPITAL FUND FOR LONDON.

### Annual Meeting of the Governors and General Council.

The annual meeting of the Governors and General Council of King Edward's Hospital Fund for London to receive the accounts and the report of the General Council for the year 1912 was held at St. James's Palace on Wednesday, April 23, the Speaker of the House of Commons being in the chair.

Those present were:—The Rev. W. L. Watkinson, D.D.; Lord Farquhar; the Chairman of the County Council (Mr. Cyril Cobb); the President of the Royal College of Surgeons (Sir Rickman J. Godlee, Bart.); the Rt. Hon. Thomas Burt, M.P.; the Rt. Hon. Sir Vezey Strong; Sir Henry Burdett; Sir Horace Marshall; Sir William J. Collins; Sir Cooper Perry; Sir John Craggs; Sir John Tweedy; Mr. Frederick M. Fry (Hon. Secretary); Mr. J. Danvers Power; Mr. Robert Fleming; Dr. Edwin Freshfield; Mr. John G. Griffiths (Hon. Secretary); Mr. Algernon E. Sydney.

In the absence of Lord Rothschild (the Hon. Treasurer) Sir John Craggs presented the account of receipts and expenditure and the balance sheet for the year ending December 31, 1912, the adoption of which was formally moved by the Speaker of the House of Commons. The resolution, having been seconded by Lord Farquhar, was then put and carried unanimously.

### Report of the General Council.

Mr. John G. Griffiths (Hon. Secretary) presented the draft report of the Council for the year 1912, as follows:—

### The Year's Figures.

The total receipts for the year 1912 were £179,378 17s. 7d. This sum was made up as follows: Donations, £7,450 16s. 8d.; contributions to capital, £424 10s. 10d.; annual subscriptions, £25,700 19s. 7d.; contribution of the League of Mercy, £18,000; from the Lewis Estate, £36,886 10s. 3d.; legacies (other than legacies to capital), £11,437 11s. 6d.; interest from investments, £79,278 8s. 9d.; together with £200 from the trustees of the Bawden Fund.

The amount distributed was £157,500, being the same as in 1911, apart from the supplementary distribution of £31,500 made possible in that year by Sir Ernest Cassel's gift. Of this sum the Hospitals received £151,000, while the remaining £6,500 was distributed amongst Consumption Sanatoria and Convalescent Homes taking London patients.

The total sum distributed amongst Hospitals, Convalescent Homes, and Consumption Sanatoria during the last ten years (after deducting grants to the extent of £7,409 16s. 7d. that had been allowed to lapse) is £1,300,340 3s. 5d.; since the foundation of the Fund a total amount of £1,633,416 8s. 5d. has been distributed.

During the year the amount spent on administration (including the balance of the expenses of the Out-patient Inquiry Committee) was £1 17s. 1d. per cent. of the total amount received. The corresponding percentage for the whole period since the inauguration of the Fund has been £1 6s. 2d.

His Majesty the King, Patron of the Fund, has been graciously pleased to continue his annual subscription of £1,000. Her Majesty Queen Mary, Her Majesty Queen Alexandra, His Royal Highness the Prince of Wales, and many other members of the Royal Family are liberal supporters of the Fund.

The list of contributions includes annual subscriptions of £5,000 from Mr. Walter Morrison, £3,000 from the Drapers' Company, £1,000 from Mr. William Waldorf Astor, and £1,000 from the Merchant Taylors' Company; besides a donation of £1,000 from the Goldsmiths' Company and an anonymous donation of £1,000.

The legacies received during the year included £3,000 allocated to the Fund by the executors of the late Miss Emma Brandreth, £2,199 15s. on account of the residue of the estate of the late Mr. George Henry Swonnell, £2,000 allocated by the executors of the late Mr. Hugh Alexander Laird, and £1,799 12s. 6d. on account of the residue of the estate of the late Mr. George Low.

### Comparison of Income, 1911 and 1912.

The total amount received in donations and subscriptions as compared with 1911 showed, excluding contributions to capital and the special donation of £31,500 from Sir Ernest Cassel, a decrease of £9,156. It should be remembered, however, that the receipts for 1911 included an anonymous donation of £10,000. Legacies showed a decrease of £37,139 5s., as compared with 1911, or of £38,838 8s. 6d. as compared with the average of the five years 1907 to 1911.

In view of the falling off of legacies to a point so far below the average, the maintenance of a distribution of £157,500 was only rendered possible by drawing upon reserves resulting from the receipt in recent years of exceptionally large legacies. The Council would emphasise the fact that the income from investments represents only about one-half the amount of the annual awards, and that for the maintenance of the present rate of distribution the Fund is dependent upon contributions received during the year.

### £200,000 from the League of Mercy.

The League of Mercy has this year contributed £18,000, being an increase of £1,000 over the sum contributed in 1911. During the thirteen years of its existence the League has furnished in all £188,000 to the Fund, making, with the sums distributed by the League direct to institutions outside the area of the Fund's distribution, a total contributed to Hospitals of £200,000. The Council desire to express their appreciation of the efforts of the voluntary workers who have so materially assisted the Fund and the Hospitals by the collection of this large sum.

### Applications for Grants.

Every eligible Hospital and Consumption Sanatorium which applied for a grant was inspected and reported upon by the Fund's Visitors, consisting as heretofore of one medical man and one layman in each case.

The scheme of the Convalescent Homes Committee for securing beds at Consumption Sanatoria and placing them at the disposal of London Hospitals for the accommodation of patients urgently needing country treatment has again been extended, the number of beds secured for the coming year amounting to 39 at five Sanatoria, as against 37 at four Sanatoria last year.

The annual statistical report on the expenditure of London Hospitals now deals with 107 institutions, and forms as nearly as possible a complete analysis of the expenditure of all the Metropolitan Hospitals which have adopted the revised uniform system of accounts. The reductions in cost recorded in the successive issues of these reports since the comparative figures were first prepared in 1903 now amount, in spite of the rise in prices and the continual introduction of new methods of treatment, to a total of no less than £37,400 a year.

### The Out-patient Committee's Report.

Lord Mersey and the Bishop of Stepney, who, with the late Lord Northcote, formed the Special Committee appointed to inquire into the methods prevailing in the London Voluntary Hospitals with regard to the admission of out-patients, issued their report in July last. On the principal questions involved, the substance of their findings was that the out-patient departments were not abused to any considerable extent, but were in some degree misused by overlapping with other forms of medical assistance, in particular by the treatment for minor ailments of patients able to pay small fees to general practitioners, of patients able to make provision by means of provident agencies, and of patients unable to benefit through lack of non-medical forms of assistance, which only the Poor Law could supply. They also found that the medical assistance provided was to some extent prejudiced by the want of co-ordination with other forms of charitable help, or lack of touch with the home conditions or previous history of the patients. For the remedy of these and other defects the principal recommendation of the Committee was the extension of the system of specially trained inquiry officers known as "Almoners," which is already in operation at many hospitals.

The Council commended this report to the careful consideration of the Hospitals, and asked for information as to the probable cost of carrying out the recommendations. In view of the prevailing uncertainty as to the effect of the National Insurance Act upon the working of the out-patient departments and upon the finance of the Hospitals, the Council had not at the end of the year considered it expedient to take any further step at the moment.

The Council desire to record their regret at the loss sustained by the lamentable illness and death of Lord Northcote, which deprived the Special Committee of his valuable services during the latter part of this inquiry. They desire to express to Lord Mersey and the Bishop of Stepney the thanks of the Fund for the great service they have rendered in undertaking the arduous work of the inquiry and in presenting so exhaustive and valuable a report.

### The Fund and Hospital Accommodation in London.

The Distribution Committee have carefully considered all schemes for the extension or reconstruction of hospitals which have been submitted in accordance with the request of the Council. The Fund has thus used its best endeavours to assist the Hospital authorities in ensuring that when capital expenditure of this kind is undertaken, adequate consideration is given to economy, efficiency, and the prospect of funds for future maintenance; and has particularly considered the relation of each scheme to the general question of hospital accommodation in London.

The General Council regret to record the loss by death during the year of His Grace the Duke of Fife, who was for twelve years a member of the Council, and presided over its meetings on several occasions. The Fund has also been deprived by death of the valuable services of the Rev. T. Bowman Stephenson, the Rt. Hon. Sir Joseph Dimsdale, and Sir Julius Wernher. Dr. Bowman Stephenson and Sir Julius Wernher had been members since the foundation of the Fund in 1897.

The President of the Local Government Board, in pursuance of his powers under the Act, has appointed Messrs. Deloitte, Plender, Griffiths and Co. to audit the accounts of the Fund for the year.

The Council are under great obligations to the Press for the valuable services they have rendered in the past, and continue to rely upon their kindly co-operation in obtaining for the Fund wider publicity and support.

The Speaker of the House of Commons, in moving the adoption of the report, said: My Lords and Gentlemen,—I have to move the adoption of the report. It is not usual at this meeting of the Council for the Chairman to make any lengthened observations, and I do not know that they are called for particularly. The report presents some features of disquiet, and on the other hand it has some very satisfactory features about it. It is a little too early, perhaps, to say what the effect of the Insurance Act may be upon the dispositions of our subscribers, but we trust that, as the novelty of it passes off, they may feel that the demands made upon them are not such as to prevent their continuing to subscribe to our Fund as they did before the Act became an Act. I will not detain you any longer, but I will formally move that the report be received and adopted.

Sir Horace Marshall seconded the motion, which was carried unanimously.

### The Distribution Committee and St. George's Hospital.

In the absence of Sir William Church (Chairman of the Distribution Committee) Sir John Tweedy moved the adoption of a report of the Distribution Committee upon the application from St. George's Hospital for a grant, as follows:—

The Distribution Committee have now completed their consideration of the application received from St. George's Hospital in 1912, and beg to report as follows:—

1. It will be remembered that St. George's last applied in 1910, and that in 1909 the hospital had paid to its school, on account of specific work done in respect of the treatment of patients, a sum of £1,875,* made up as follows: Salaries £1,075, cash payment £550, rent remitted £250. The departments in respect of which the payment was made included bacteriological and pathological laboratories, *post-mortems* and museum, and the payment was arrived at by an apportionment of expenses between hospital and school. As compared with this payment of £1,875,* the charge to the general account of the hospital had before 1909 been £1,000, the £250 remission of rent not appearing in the accounts, and the cash payment of £550 having been paid out of a Discretionary Fund available for school purposes.

2. The King's Fund came to the conclusion that the payment of £1,875* from the General Fund of the hospital to the school was excessive, and that, in order that the accounts of the hospital should be clear from the indirect contribution to medical education involved in an overpayment, the total should be reduced to £1,000, the balance of £875* being repaid by the school. This could readily have been done by charging the £550 and the £250 mentioned above to the Discretionary Fund instead of to the General Fund.

* Now reduced by £75 in the revised figures supplied by St. George's.

3. The Committee of St. George's Hospital, however, did not accept this decision, and they disputed the conclusion of the Distribution Committee on the question of fact. That conclusion had been based on comparative figures obtained from a number of important hospitals, which showed that at St. George's the cost per bed to the hospital of the departments in question exceeded the cost to the hospital of similar departments at other hospitals by an amount which, in the opinion of the Fund, could not be justified by the interests of the patients as distinct from those of the school.

4. St. George's subsequently, themselves, obtained figures from several hospitals, and submitted a statement to the late Lord Mayor (who was making a similar inquiry as President of the Hospital Sunday Fund), upon which he pronounced the opinion that the cost at St. George's was not excessive and did not represent a larger cost per bed than was the case at many other London hospitals. When considering the present application for a grant from the King's Fund, the Distribution Committee obtained from St. George's a copy of this statement. The Committee have made a careful examination of this fresh evidence, and have corresponded both with St. George's and with the other hospitals upon the subject; and they regret to find that this examination does not enable them to modify their previous recommendation.

5. It is clear from their detailed inquiries that the conclusion of the late Lord Mayor was chiefly based on imperfect information as to what work was covered by the figures of the other hospitals. The payment of £1,800 at St. George's covered bacteriological and pathological examinations, *post-mortems* and museum. But the statement laid before the Lord Mayor, compared figures for St. George's, which *excluded* the cost of *post-mortems* and museum, with figures for other hospitals which *included* the cost of *post-mortems* in every case, and of the museum in the rare instances in which the museum is not wholly paid for by the school. In the case of St. George's the amount excluded (as given in another part of their statement) was no less than £750 out of the total paid to the school, and the figure which was brought into comparison with the other hospitals was thus £1,050 instead of £1,800.

6. When all the departments in question are taken into account, and when certain minor corrections are made, it appears that even in the case of the five hospitals whose figures are used both by St. George's and by the Fund the cost per bed at St. George's still very largely exceeds the cost of the others, while if six other hospitals whose figures have been used by the Fund are also taken into consideration the cost at St. George's works out at about two and a quarter times the average. The Distribution Committee do not attach importance to the exact ratio except as evidence that the excess is very large.

7. In justification of their expenditure St. George's have argued: first, that the work done in the departments in question differs from that at other hospitals in quantity and quality; and, second, that skilled investigators are necessary for the efficient treatment of the patients as well as for the purposes of the school, and as the school cannot afford to pay as much as at other hospitals, the hospital is compelled to pay a larger share of the cost.

8. On the first point the evidence brought forward by St. George's does not convince the Committee that the value of the work done in their bacteriological and pathological laboratories in the sole interests of the patients exceeds that at other hospitals; and, moreover, even if the argument were proved in regard to these departments it would not justify the heavy expenditure on the museum which with the *post-mortems* accounts for the greater part of the excess cost.

9. The second argument assumes that the school has not sufficient resources from which to meet its due share of the cost, and ignores the existence of the Discretionary Fund, which is, at the direct wish of the donors, available to meet the legitimate expenses of the school as distinct from the hospital. The amounts so available at St. George's during the past few years have exceeded £2,000 a year, while the amount chargeable to the Discretionary Fund would have been only £800.

Conclusion.

10. Under these circumstances it still appears to the Distribution Committee that the existing apportionment between hospital and school of the cost of the departments in question at St. George's involves a charge to the hospital very largely in excess of the proportionate cost of similar work at other hospitals; that the excess is greater than can be justified by the interests of the patients as distinct from those of the school; and that so long as this is the case the accounts of St. George's cannot be held to be clear from any payment in respect of medical education.

11. The Distribution Committee therefore regret that they see no reason why the Fund should depart from its previous conclusion, viz.: that the apportionment should be revised, and that the charge to the hospital, which in 1909 amounted (on the amended figures now supplied by St. George's) to £1,800, should be reduced by £800, this excess being repaid by the school.

12. The Distribution Committee would emphasise the facts: (*a*) That previous to 1909 the accounts of St. George's Hospital showed a total charge to the hospital of only £1,000; (*b*) that in the statement submitted by St. George's to the late Lord Mayor the total brought into comparison with the totals at other hospitals was only £1,050; (*c*) that the sum arrived at by the Fund as the proper proportion to be charged to the hospital was and still is £1,000; and (*d*) that the greater part of the excess of £800 is due to expenditure on the museum, which at almost every other hospital is wholly borne by the school.

13. The Distribution Committee would also point out: (i) That the sum of £1,000 leaves a sufficiently wide margin above the average to allow for such differences between one hospital and another as may be called for in the interests of the patients; (ii) that the authorities of St. George's have at their disposal a Discretionary Fund sufficient to enable the full amount of the school proportion of the annual expenditure (under the revised apportionment) to be paid for by the school out of moneys earmarked by the donors as available for school purposes; (iii) that it is thus possible for St. George's, without hampering the work either of the hospital or of the school, to make the required reapportionment, and clear the accounts of the hospital from any contribution towards medical education; and (iv) that there should be no insuperable obstacle against the acceptance by St. George's of this decision, since the Fund has at no time cast any doubt upon the *bona-fides* of those whose conclusions on this matter they have reluctantly found themselves unable to accept.

The motion, having been seconded by Sir Cooper Perry, was put, and after discussion carried unanimously.

Request by St. George's Hospital for an Independent Inquiry.

In the absence of Lord Bessborough (Chairman of the Executive Committee), Sir William Collins moved the adoption of a report of the Committee upon a request from St. George's Hospital for an independent inquiry. Sir William said that this report practically repeated the recommendation of the Committee at the last meeting that they did not consider that the question was one which required the application of exceptional measures of a kind which did not appear to be contemplated by the Act incorporating the Fund. It meant that the opinion of the Executive stood, viz. that it would be unwise to take this matter from the existing reference to the Distribution Committee, and also that it would be undesirable to set up an exceptional form of arbitration, as this would amount to an abdication of the functions of the Council, which under the Statute was the final body to decide upon matters relating to the distribution of the Fund.

The adoption of the report was seconded by Dr. Freshfield, and carried with one dissentient.

On the motion of Sir William Collins, seconded by Sir Vezey Strong, a resolution was passed on the question of rules and regulations.

On the motion of the Chairman of the County Council (Mr. Cyril Cobb), seconded by the President of the Royal College of Surgeons (Sir Rickman J. Godlee), a vote of thanks to the Speaker for presiding was carried unanimously.

The Speaker having briefly acknowledged the vote of thanks, the proceedings terminated.

Mr. J. Danvers Power has since addressed the following letter to the Speaker :—

Dear Mr. Lowther,—Although I personally agree with King Edward's Hospital Fund that the facts connected with payments made by St. George's Hospital to its medical school are such as to disqualify it from grants from any Fund which is precluded from contributing to medical education. Yet, as the Hospital Sunday Fund takes, and acts upon, a contrary view, I feel that the only right and prudent course is to comply with the request of St. George's Hospital for an independent judgment, by which it, the hospital, will abide.

In my opinion it is necessary to do this no less in the interests of the King's Fund than in those of the hospital itself. As, however, my motion to that effect at the Council meeting this morning, of which full notice had been given, did not even attract a seconder, it is evident that my idea of what is both fair and polite entirely differ from those of my colleagues. And as I have the misfortune, therefore, to be unable to defend the justice of the decision arrived at and its consequent serious effects on the hospital, I desire, without intending any disrespect for so distinguished a body, to resign my seat on the Council at the earliest opportunity which will be legal under the Act.—Yours faithfully, J. Danvers Power.

Oxford and Cambridge Club, April 23, 1913.

